# Benefit-Seeking or Risk-Taking? Examining the Portrayal of Cosmetic Surgery in Chinese News, 2000–2019

**DOI:** 10.3390/ijerph18010048

**Published:** 2020-12-23

**Authors:** Shaojing Sun, Jinbo He, Xiaohui Yang, Fan Wang

**Affiliations:** 1 School of Journalism, Fudan University, Shanghai 200433, China; ssun@fudan.edu.cn (S.S.); 18210130009@fudan.edu.cn (X.Y.); 2 School of Humanities and Social Science, Chinese University of Hong Kong, Shenzhen 518172, China; 3 Department of Politics, East China Normal University, Shanghai 200241, China

**Keywords:** cosmetic surgery, Chinese media, framing, health risk, content analysis

## Abstract

Analyzing 311 news articles published in representative Chinese newspapers, this study investigated how cosmetic surgery (CS) was represented in Chinese media from 2000 to 2019. Employing a coding scheme based on prior literature and sampled data, the study analyzed both features of the articles and profiles of the patients in the media. Results showed significant differences in media reporting across issue categories, reporting frames (benefit-focused, risk-focused, neutral), sources of information, drivers for and impacts of having surgeries. Among all the reasons for obtaining CS, boosting career confidence was ranked at the top. Additionally, significant differences in reporting were observed across types of the newspapers, gender of the patients, and time periods of the coverage. Implications of the findings for health promotion and regulation were discussed in reference to the social, cultural, and media context in China.

## 1. Introduction

Cosmetic surgery (CS) refers to any invasive procedure with patients’ primary intention to achieve a more desirable appearance. The term has been loosely defined and often is used interchangeably with jargon such as plastic surgery and reconstructive surgery. Dean et al. argued for a holistic and contextual view of CS, emphasizing its nuanced, lasting and complex consequences. Considering the significant impacts of undertaking CS on a patient—both positive and negative, visible and invisible—we reason that a definition highlighting both the medical and psychological effects may help the public form a more comprehensive and sober view of having CS [1]. As such, CS can be viewed as a medical as well as a psychological intervention or, at a minimum, a surgical procedure with both physical and psychological consequences.

According to the report by the International Society of Aesthetic Plastic Surgery (ISAPS) in 2018, cases of cosmetic surgery have been growing at an annual rate of 5.4% across the world. More than 23 million cosmetic surgery procedures were performed in 2018, with over 55,000 cases involving teenagers [2]. The number of CS cases in China reached 20 million in 2018, surpassing the United States and topping the world [3]. Furthermore, the growing Chinese market has led to a booming international cosmetic surgery tourism industry [4]. In China, popular surgical procedures include double-eyelid operation, breast augmentation, jaw reshaping, and others. The average age of CS customers in China is about 24.45 years old, with those born in the 1990s making up the largest consumer group [5].

CS is not merely a medical phenomenon, but rather it is embedded in a rich social, cultural, and historical context. As Elliott contended, the upsurge of CS in the West is driven by both the macroscopic and microscopic changes in culture, media, and interpersonal relations [6]. For instance, the celebrity-inspired surgical culture and media representations have given rise to a shift away from the public focus on celebrity personalities to their body parts. In contrast, factors affecting Chinese women’s craving for CS are diverse, including but not limited to, cultural values (e.g., Confucianism, consumerism) and media coverage [7].

There is ample evidence suggesting that media exposure plays a central role in forming individuals’ attitudes toward CS [8,9]. Research has shown that Western media coverage of CS tends to emphasize ideal beauty achievement, ease of procedures, and social/physical/mental benefits—but tends to understate the associated risks [10,11]. Consider, for example, the popular Cosmetic Surgery Makeover TV programs. Crockett et al. found that patients who regularly watched such programs were more likely to pursue cosmetic surgeries than were lighter viewers [12]. Nabi, furthermore, emphasized that the increasing demand for CS, at least in part, results from the ease of access to related information through media [13].

The way media reports and represents CS speaks to the well-established framing theory. Framing means that certain aspects of reality are selected, presented, and emphasized to make them more noticeable, meaningful, and memorable in a communication text [14]. Media framing reflects the positions, principles, and preferences of news producers. Through framing, news media can exercise a significant impact on shaping how people think and talk about certain issues, particularly controversial ones. Regarding CS, media coverage could frame the procedure by highlighting its beneficial features, or conversely, by highlighting its inherent risks. Such different angles of framing are likely to elicit different views of CS. In essence, media frames “promote a particular problem definition, causal interpretation, moral evaluation, and/or treatment recommendation” [14]. For instance, Brooks analyzed four American magazines (e.g., Vogue, 2001–2003) and identified two major narrative frameworks which either presented CS as a new technology or as patients’ candid accounts of positive experiences with CS [15]. Adams found that American newspapers (from 2000 to 2007) tended to downplay risks associated with CS [16]. Furthermore, media normalized CS as a safe and effective route to bolster self-confidence and remain competitive on the job market. Woodstock observed both positive and negative frames in reporting CS in mainstream women’s magazines in America [17]. The positive frames linked having CS to maintaining youth and beauty, whereas the negative ones linked it to vanity and violence. There are a variety of symbolic and communicative components—such as quoted sources of information, metaphors—that can be used to construct a particular media frame. Among all possible factors, quoted information sources have been widely studied and viewed as a crucial component in constituting media frames. Indeed, past research has shown that media’s selection and usage of different information sources can affect audience’s perceptions of and attitudes toward a particular issue covered by the media. For instance, Imison and Schweinsberg found that patients were quoted and featured widely in Australian TV and newspaper coverage of cosmetic surgery. Such quotes and features, the authors argued, help build personal relevance to the audiences and comport with the centrality of human interest in general news reporting [18]. Wen et al. noted that while expert sources were used more frequently in YouTube videos about CS, the presence of typical-consumer sources in the videos generated much stronger viewer interest in CS [19].

The divergences in public aesthetic tastes, cultural traditions, and social systems may lead to distinct media representations of CS. Consider the fact that Chinese media, over the past decades, have been facing pressure to toe the political lines and to increase revenues. As a result, Chinese media have largely branched into two camps: the government-sponsored and the market=oriented. Government-sponsored media (e.g., The People’s Daily), relatively free of the pressure for making revenues, tend to put more emphasis on “hard” news (e.g., politics and government policies), whereas market-oriented media tend to report more on “soft” news (e.g., social events, entertainment) so as to accrue its readership and grow revenue [20]. The different media orientations could lead Chinese media to approach and frame CS differently, as such framing may carry political, cultural, economic implications.

Past research has shown that gender-specific body ideals in media may influence males’ and females’ body dissatisfaction in different ways (e.g., drive for masculinity for males vs. drive for thinness for females) [21]. Furthermore, gender differences in media portrayal could affect males’ and females’ attitudes toward CS differentially. For instance, recently, Wen et al. reported that media had both direct and indirect effects on Singaporean young peoples’ attitudes toward CS, with men being more susceptible to media influence than women [22]. As such, it is of interest to examine how patients (e.g., males vs. females) were represented in the media.

In light of the above literature, departing from media framing theory, we propose the following research questions:

RQ1: How did Chinese media frame CS (e.g., themes, angles, information sources) in general?

RQ2: Did Chinese government-sponsored media and commercial media frame CS differently?

RQ3: How were CS patients represented in Chinese media?

RQ4: How did Chinese media’s framing of CS evolve across time (2000–2009 vs. 2010–2019)?

Below, we describe our research data and introduce the content analysis procedure; next, we explain our research findings in accordance with the order of the research questions; finally, implications and conclusions of the findings are presented.

## 2. Materials and Methods

### 2.1. Sample of Data

**Figure 1:**
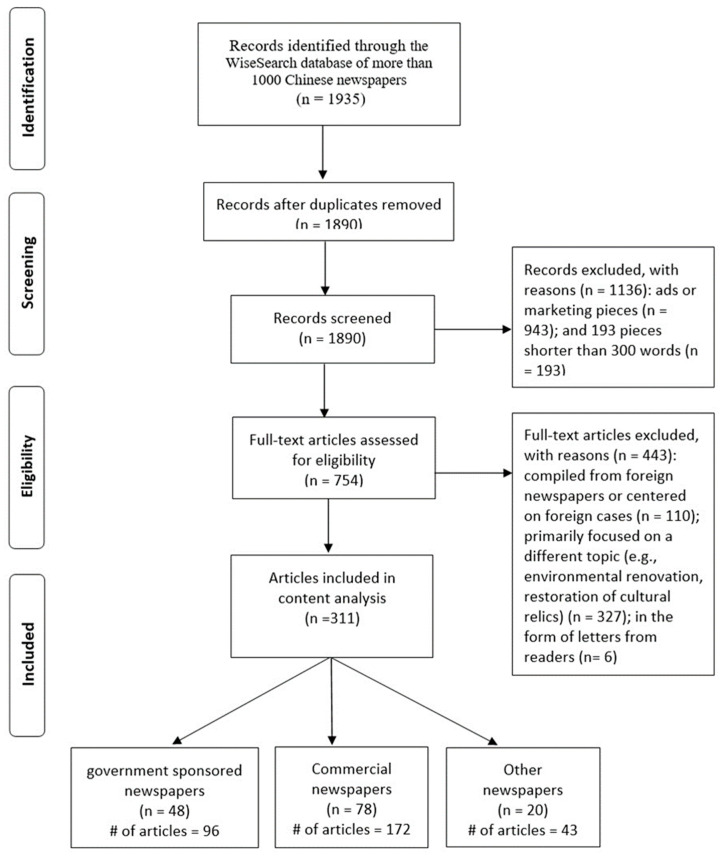
PRISMA flowchart of article inclusion. #. number of selected articles.

A preliminary search of the keyword CS in Chinese TV and radio news scripts turned up very few results. Therefore, we mainly focused on Chinese newspapers for the present study. Articles published in Chinese newspapers (Jan. 1, 1998—Dec 31, 2019) were retrieved from the WiseSearch database, using the following string of search terms: “整容 OR 整形,” which literally translate to “cosmetic surgery OR plastic surgery.” After screening out duplicate and irrelevant articles, a total of 311 news articles were retained and subjected to final content analysis. The PRISMA diagram (Figure 1) presents the detailed information about our strategies for article searching and screening.

### 2.2. Strategies of Analysis

The authors reviewed past literature on coding Chinese newspapers [23,24,25], read sample articles, and created a coding scheme together. Basic information about the articles included newspaper type (government-funded, commercial, other), publication time, type of article (e.g., editorial, news), article length, and page section of placement (e.g., health section of a newspaper). A newspaper was classified as “other” if it showed neither a clear market-orientation nor a government sponsorship. These “other” newspapers were primarily local or specialized (e.g., focusing on technology), and tended to have shorter publication histories and lower circulations.

In addition to article type, the authors coded the contents by examining types of CS (general vs. specific), issue categories or focal issues (e.g., exemplars/cases of patients, litigation/lawsuits, safety and risk concern, popularity of CS), reporting angles (benefit-focused, risk-focused, neutral), types of information sources (experts, patients, other), drivers for CS (help career development, pursue domestic ideal of beauty, influence by friends), and impacts of CS (physical, psychological, social). Following the same procedure created by Shepherd and Seale [26], we separately coded individual profiles of the patients reported in the news articles. A total of 215 profiles of CS patients were identified and subjected to content analysis. For patient profiles, we coded the types (breast surgery, fat suction, nose surgery, eyelids) and the impacts (physical, psychological, relational impacts) of surgeries, explanations for surgical failures, and mentioned penalties for hospitals due to the failures.

The authors randomly selected 40 articles for piloting and calculating intercoder reliability. With the finalized coding scheme, the intercoder reliability estimates (Krippendorff’s alpha) for each coding category ranged from 0.79 to 0.95. Any discrepancy of coding was discussed and resolved between the coders.

## 3. Results

### 3.1. General Findings

**Figure 2:**
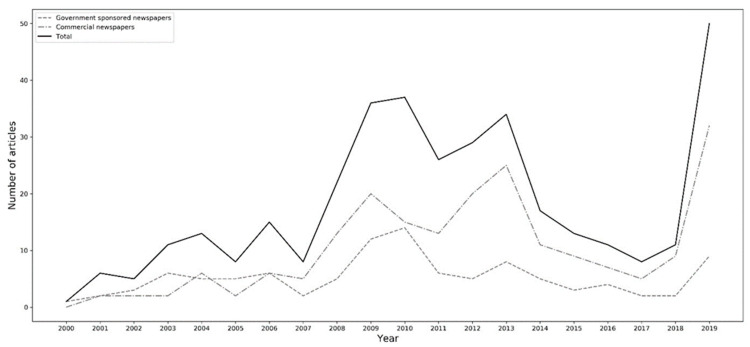
Plot of articles across time.

Figure 2 presents the general trajectory of media coverage of CS. Overall, the coverage showed a slight upward trend of media reporting over the past two decades. There was no statistically significant difference between the types of surgeries covered in the media. About 52% of the articles mentioned specific types of surgeries (e.g., breast augmentation, eyelid operation), and 48% mentioned CS in general. Among the covered issues, safety and risk concern accounted for the highest share—about 32% of the sampled articles—followed by the issue of exemplar cases, accounting for 23%. There were statistically significant differences between reporting frames, with risk-focused frames being ranked higher (36%) than benefit-focused (22%) and neutral frames (11%). Medical experts were the most cited information source, appearing in more than 70% of the articles. In contrast, about 52% of the articles quoted patients. The main drivers for CS comprised boosting self-esteem/confidence, accounting for 61% of the articles, and the impact of media or friends, accounting for about 16%. As for the impact of CS, the most frequently discussed were physical impact (65%) and psychological impact (32%).

### 3.2. Comparisons by Newspapers

As shown in Table 1, commercial newspapers were more likely to report specific surgeries than were government-funded ones (χ^2^ = 18.56, *p* < 0.001). Moreover, commercial newspapers were more likely to report the exemplars of patients. Government-funded newspapers were more likely to use risk-focused frames (χ^2^ = 9.47, *p* = 0.002). The difference in employing benefit-focused frames was only marginally significant across newspapers (χ^2^ = 4.30, *p* = 0.038). Results also showed that commercial newspapers were more likely to discuss the impacts of CS—particularly physical (*p* = 0.012) and psychological impacts (*p* = 0.037)—than government-sponsored media.

### 3.3. Comparisons between Male and Female Patients

As shown in Table 2, there were a total of 215 exemplar patient-cases presented in the newspapers. Among the specific surgeries, eyelid operation appeared most frequently (17%), followed by nose surgery (9%). The media tended to discuss the failures of the patients’ surgeries (42%) more often than the successes (29%), with χ^2^ = 10.56, *p* < 0.001. Whether it is about success or failure, the media tended to discuss more the surgeries’ physical impacts on patients than the psychological and relational impacts (χ^2^ = 27.29, *p* < 0.001; χ^2^ = 59.79, *p* < 0.001). Of note, only about 25% of the articles mentioned potential reasons for the failures of CS. Additionally, about 15% of the articles mentioned punishments for the hospitals where the surgeries were performed.

Of the 215 patient-cases presented in the newspapers, male and female patients accounted for a share of 16.7% and 82.3%, respectively. As for gender differences, more surgical successes were reported for male patients (χ^2^ = 10.72, *p* < 0.001), while more surgical failures for female patients (χ^2^ = 7.86, *p* = 0.005). Regarding the successes of CS, the newspapers discussed more frequently the physical impacts on men than on women (χ^2^ = 8.36, *p* = 0.002). In contrast, regarding the failures, the media discussed the physical and psychological impacts on women more frequently than that on men (χ^2^ = 8.35, *p* = 0.003; χ^2^ = 7.34, *p* = 0.006).

### 3.4. Comparisons between 2000–2009 and 2010–2019

As shown in Table 3, comparing the past two decades, results showed that the media were more likely to report CS in general in the recent decade than 10 years ago (χ^2^ = 6.99, *p* = 0.008). There was a slight decrease in using benefit-focused reporting frames over the years (χ^2^=4.96, *p* = 0.025), though there was no significant change in harnessing risk-focused frames. Newspapers tended to quote patients less frequently (χ^2^ = 5.13, *p* = 0.023), but to quote medical experts more frequently in recent 10 years than in the previous decade (χ^2^ = 4.73, *p* = 0.029). During the first decade, more articles emphasized boosting self-confidence as a reason for undertaking CS (χ^2^ = 4.09, *p* = 0.041), but fewer articles mentioned pursuing beauty ideals as the main reason (χ^2^ = 6.35, *p* = 0.011). Although media coverage of the physical impacts of CS was prevalent in both decades, there was a statistically significant increase over time (χ^2^ = 6.03, *p* = 0.014).

## 4. Discussion

The present study indicates that CS has only received limited attention from Chinese newspapers, in spite of a slight growth over the past decade. On the whole, media articles about CS are relatively brief, scattered, or even tabloidized.

Regarding RQ1, not surprisingly, Chinese media expressed relatively high concerns about the safety and risk of having CS. Our finding contrasts with the results by Pitts–Taylor showing that media tend to frame CS procedures as easy and quick solutions to one’s body concern, while downplaying its risks [27]. Such a finding comports with the high rate of accidents and lawsuits due to unsuccessful CS in China. According to the statistics released by the China Consumers Association, on average, there were more than 20,000 lawsuits filed by Chinese consumers each year because of CS failures [28]. In contrast, the rates of surgical mishaps and accidents were much lower in developed countries. As such, the frequent usage of risk-focused frames by Chinese media testify to the fundamental roles the media can play in alerting consumers, governors, and general public to the risks.

In building frames, Chinese media tend to rely more on quoting experts (72%), as well as patients (52%). Interestingly, the usage of frames and information sources seem to vary across media and culture. For instance, Cho found American TV networks new coverage put an emphasis on the risks associated with CS, and it relied extensively on experts from other fields in its reporting [29]; Wen et al. found that nearly 60% of YouTube videos promoted CS by emphasizing its beneficial effects, while only 9% emphasized its risks [19].

Regarding RQ2, we did find significant differences in coverage across different types of media. Commercial newspapers tend to present more patients’ exemplars than do government-sponsored media. Constructing exemplars or narratives has long been viewed as a communication strategy to appeal to audiences, as exemplars can help improve the vividness, coherence, and appeal of a message [30]. Furthermore, commercial newspapers tended to emphasize more on the benefits, whereas the government-sponsored newspapers tended to stress more on the risks. Again, such a discrepancy could be due to the different orientations of the media—either to prioritize government agenda or to prioritize business interests. It is well known that despite being under the ruling of the same media system, commercial media in China put more emphasis on expanding its readership and growing its revenue, and hence are more likely to rely on advertising and marketing. Moon reported that Korean media frequently promoted particular clinics or CS surgeons in subtle or unperceivable ways [31]. We reason this also is the case in China, as we did observe that concrete names of certain clinics and surgeons appeared in the articles.

More interestingly, among all the reasons for obtaining CS, boosting career confidence was ranked at the top, followed by the impacts of friends, and the drivers for beauty ideals were the least frequently mentioned. As noted by Hua [32], “Being Good-Looking Is Capital” in today’s China. Facing fierce competition, job seekers in China have to pull out all means to acquire a decent job; consequently, more people view physical appearance as a leverage and hence turn to CS to increase their capital for success [32,33]. However, this finding contrasts with those identified in Western cultures, where media idols or celebrities serve as body ideals driving consumers for having CS [6]. Our study shows that Chinese consumers go for CS mainly out of the reason for career development, but not for achieving media-rendered ideals. Body beautification, in this case, serves more instrumental causes than ritualistic ones (e.g., obsession with a media ideal).

As for RQ3, the finding—that the majority of the patient-cases reported in the media were female (86.3% of the total cases)—is in line with the current male-to-female ratio of CS consumers in China [3]. A possible explanation is that Chinese domestic employers are much more likely to set expectations for women’s appearances in their job advertisements [34]. As Zhang explained, there exists “beauty oppression” in contemporary Chinese society, where beauty functions as both a source of social capital and a shackle of free spirit for Chinese women [7]. The high percentage of surgical failures for women—as compared to men—draws attention to the complex and diverse surgical procedures Chinese women have undergone. The thriving CS industry in China comes with enormous risks, due to inadequate supervision and lax regulations [35,36]. To date, there are only about 17,000 licensed cosmetic doctors in China, while illegal practitioners in this field have exceeded 150,000. The whole industry also suffers the use of smuggled drugs and materials [37]. The present study has shown that Chinese media coverage paid more attention to physical impacts of CS as compared to emotional and relational impacts. Such a discrepancy could be due to the low recognition of emotional and mental health in China. By contrast, emotional impacts of CS are more likely to be acknowledged and reported in Western culture. For example, Adams interviewed American patients having CS, and the patients reported they expected that the surgeries would bring them both physical and psychosocial changes (e.g., enhancing self-esteem) [38]. Polonijo and Carpiano, analyzing popular women’s magazines circulating in Canada, observed that CS in general was framed as a risky but worthwhile option for women to improve their physical appearance and emotional health [39].

AS for RQ4, comparing the two decades, we observe a slight decrease of using the benefit-focused frames across time, though no significant difference in using the risk-focused frames. Such a trend corresponds to the growing accidents caused by having CS in recent years. Concerns about the safety and risk of having CS speak to the nature of new technologies such as CS. As Shin and Hwang noted, the affordances of new technologies (e.g., security) link to users’ trust and satisfaction [40]. Some of those relatively invasive surgical procedures (e.g., breast surgery) carry inherent risks. Such risks will continue to be in the spotlight of media, along with the development of new technologies for beautification.

Several limitations and caveats should be noted. First, although we intended to explore Chinese media coverage of CS, the present study only focused on Chinese newspapers. Magazines and online news, which could potentially feature CS and hold great research value, were not examined. Second, a mere content analysis cannot fully reveal how the meanings and images of CS are constructed in a culture. Future research should employ multiple methods (e.g., interviews and survey), multiple populations (e.g., surgeons, patients), and multiple media platforms (e.g., social media, business websites) to explore social implications and ramifications of cosmetic surgery. In all, this study provided insight into the current state of affairs about how Chinese media cover CS. Improving public awareness of the potential risks of doing CS should be at the top agenda of both health experts and media professionals. Furthermore, scientific and effective regulations of monitoring and managing those risks should be established, enforced, and evaluated for the benefits of public health.

## 5. Conclusions

Overall, this study showed that safety and risk concern was the major issue Chinese media focused on while reporting CS. Chinese media tended to frame CS more frequently in terms of its risks as opposed to its benefits. Furthermore, experts were more often quoted as compared to other information sources. Commercial newspapers’ reporting emphasized more the benefits, and less the risks, of having CS than government-sponsored media.

A comparison of our study and prior research shows that the construction and application of message frames about CS vary across media and culture. Western media—with women’s magazines in particular—tended to cast CS in a more favorable light. Chinese newspapers put more emphasis on the risks and concerns about undergoing CS. Psychological and relational impacts of, both successes and failures, undertaking CS, received relatively less attention from media.

There was a higher percentage of media-reported successes, while a lower percentage of media-reported failures, of having CS for males than for females. The recent decade, compared to the previous decade, saw a decrease of using benefit-focused frames in media coverage. Future studies should investigate how new media platforms, particularly social media, frame CS and the potential consequences of such framing.

## Figures and Tables

**Table 1:** Newspaper reporting on evidence, definition, etiology, causes, and consequences of cosmetic surgery, overall and by newspaper genre.

	Overall		Government-Funded (n = 96)		Commercial (n = 172)		
	n	% (95% CI)	n	% (95% CI)	n	% (95% CI)	$x^{2}\;p\mathrm{Value}$
Type of surgeries		$x^{2}$(1) = 3.02					
General surgery	149	47.9 (39.2–50.2)	47	49.0 (38.8–59.1)	71	41.3 (33.9–48.6)	0.277
Specific surgery	162	52.1 (46.6–57.9)	41	33.3 (53.3)	121	56.3 (49.8–62.6)	<0.001
Issue category		$x^{2}$(3) = 27.34 ***					
Exemplars or patients	70	22.5 (18.0–27.3)	11	11.5 (5.2–18.6)	59	27.4 (21.5–34.0)	<0.001
Litigation/lawsuits	52	16.7 (12.9–20.9)	22	22.9 (14.6–31.9)	25	14.5 (9.7–20.2)	0.118
Safety and risk concern	98	31.5 (26.4–36.7)	31	32.3 (23.5–41.3)	53	30.8 (23.7–37.6)	0.910
Popularity of CS	51	16.4 (12.5–20.9)	20	20.8 (13.8–29.5)	25	14.5 (9.4–19.9)	0.249
Reporting frames		$x^{2}$(2) = 58.22 ***					
Benefit-focused	69	22.2 (17.7–27.0)	12	12.5 (6.7–19.4)	41	23.8 (17.4–30.9)	0.038
Risk-focused	113	36.3 (30.5–41.8)	48	50.0 (45.6–57.8)	52	30.2 (26.6–34.1)	0.002
Neutral/both	33	10.6 (7.4–14.1)	13	13.5 (7.1–20.8)	16	9.3 (5.5–14.3)	0.386
Source of information		$x^{2}$(2) = 237.79 ***					
Patients	161	51.8 (46.3–57.2)	45	46.9 (36.8–57.3)	90	52.3 (44.9–60.0)	0.466
Experts	224	72.0 (67.2–76.8)	65	67.7 (58.2–77.2)	127	73.8 (67.3–80.5)	0.354
Other (e.g., celebrity)	36	11.6 (7.7–15.1)	11	11.5 (5.5–18.5)	19	11.0 (6.4–16.2)	0.996
Driver for surgery		$x^{2}$(2) = 256.23 ***					
Career/confidence	189	60.8 (55.3–66.2)	54	56.3 (46.1–66.7)	105	61.0 (53.8–68.1)	0.524
Domestic ideal of beauty	21	8.4 (5.5–11.6)	8	8.3 (3.4–14.6)	12	7.0 (3.4–11.2)	0.871
Friends	51	16.4 (12.2–20.6)	18	18.8 (11.2–27.4)	26	15.1 (9.9–20.4)	0.549
Mention the impact of CS		$x^{2}$(2) = 225.29 ***					
Physical impacts	203	65.3 (59.8–70.1)	59	61.5 (51.0–70.5)	132	76.7 (71.1–81.3)	0.012
Psychological impacts	100	32.2 (26.7–37.0)	23	24.0 (15.3–32.3)	64	37.2 (32.7–41.3)	0.037
Social impacts	25	8.0 (5.1–11.3)	8	8.3 (3.2–14.3)	13	7.6 (3.7–11.7)	0.996

Note. *** *p* < 0.001.

**Table 2:** Newspaper reporting on patient profiles.

	Overall (n = 215)		Male (n = 36)		Female (n = 179)		
	n	% (95% CI)	n	% (95% CI)	n	% (95% CI)	$x^{2}$*p* Value
Type of surgeries		$x^{2}$(3) = 23.01 ***		$x^{2}$(3) = 12.71 **		$x^{2}$(3) = 16.6 ***	
Breast surgery	14	6.5 (2.6–8.8)	0	0	12	6.7 (3.2–10.6)	0.229
Fat suction	9	4.2 (1.8–7.2)	0	0	9	5.0 (2.2–8.3)	0.358
Nose surgery	20	9.3 (5.6–13.3)	2	5.6 (0.0–16.8)	18	10.1 (6.0–14.6)	0.593
Eyelids	36	16.7 (11.9–22.1)	6	16.7 (4.9–29.7)	30	16.8 (11.4–22.5)	0.995
General impacts of surgeries		$x^{2}$(2) = 10.56 **		$x^{2}$(2) = 9.75 **		$x^{2}$(2) = 21.79 ***	
Surgical successes	62	28.8 (23.1–34.9)	19	52.8 (36.4–70.3)	43	24.0 (18.0–30.9)	<0.001
Surgical failures	90	41.9 (34.9–48.6)	7	19.4 (7.1–31.4)	83	46.4 (38.9–53.8)	0.005
None	63	29.3 (23.5–35.5)	10	27.8 (13.3–43.2)	53	29.6 (22.9–36.4)	0.984
Impacts of surgical successes		$x^{2}$(2) = 27.29 ***		$x^{2}$(2) = 12.04 **		$x^{2}$(2) = 17.99 ***	
Physical impacts	60	27.9 (22.2–34.0)	18	50.0 (33.3–68.7)	42	23.5 (17.6–29.9)	0.002
Psychological impacts	35	16.3 (11.9–21.2)	7	19.4 (7.7–33.3)	28	15.6 (11.2–21.4)	0.750
Relational impacts	19	8.8 (5.5–12.5)	6	16.7 (5.4–30.6)	13	7.3 (3.6–11.4)	0.136
Impacts of surgical failures		$x^{2}$(2) = 59.79 ***		$x^{2}$(2) = 6.75 *		$x^{2}$(2) = 56.02 ***	
Physical impacts	85	39.5 (32.8–46.0)	6	16.7 (5.3–29.0)	79	44.1 (36.5–51.8)	0.003
Psychological impacts	45	20.9 (15.6–26.7)	1	2.8 (0.0–9.1)	44	24.6 (18.2–31.2)	0.006
Relational impacts	18	8.4 (4.7–12.1)	1	2.8 (0.0–9.1)	17	9.5 (5.3–14.0)	0.318
Explain reasons for failures	54	25.1 (19.5–31.2)	4	11.1 (2.3–21.9)	50	27.9 (21.6–34.4)	0.054
Penalty of hospitals	32	14.9 (10.4–20.0)	3	8.3 (0.0–18.5)	29	16.2 (10.9–21.6)	0.340

Note. *** *p* < 0.001, ** *p* < 0.01, * *p* < 0.05.

**Table 3:** Newspaper reporting on cosmetic surgery across time.

	2000–2009 (n = 125)		2010–2019 (n = 186)		
	n	% (95% CI)	n	% (95% CI)	$x^{2}\;p\mathrm{Value}$
Type of surgeries		$x^{2}$(5) = 39.97 ***		$x^{2}$(5) = 170.41 ***	
General surgery	44	35.2 (27.0–43.2)	95	51.1 (43.5–59.0)	0.008
Specific	70	56.0 (46.5–64.4)	92	49.5 (42.1–56.8)	0.309
Issue category		$x^{2}$(3) = 7.75 *		$x^{2}$(3) = 22.45 ***	
Exemplars or patients	24	19.2 (11.9–26.2)	46	24.7 (18.6–30.7)	0.314
Safety and risk concern	37	29.6 (21.2–37.9)	61	32.8 (26.1–39.5)	0.638
Regulation/lawsuits	25	20.0 (13.5–27.3)	27	14.5 (9.4–19.7)	0.265
Popularity of CS	20	16.0 (9.9–22.2)	31	16.7 (11.5–22.0)	0.983
Reporting frames		$x^{2}$(2) = 24.75 ***		$x^{2}$(3) = 39.19 ***	
Benefit-focused	38	30.4 (26.6–35.2)	35	18.8 (13.3–24.6)	0.025
Risk-focused	42	33.6 (25.5–41.6)	71	38.2 (31.6–45.5)	0.483
Neutral/both	11	8.8 (4.2–13.7)	22	11.8 (7.0–16.7)	0.507
Source of information		$x^{2}$(2) = 92.14 ***		$x^{2}$(2) = 174.76 ***	
Patients	75	60.0 (51.3–68.5)	86	46.2 (38.8–53.6)	0.023
Experts	85	68.0 (59.1–75.6)	148	79.6 (73.3–86.9)	0.029
Other (e.g., celebrity, activist)	15	12.0 (6.7–17.7)	21	11.3 (7.0–16.2)	0.991
Driver for surgery		$x^{2}$(2) = 133.39 ***		$x^{2}$(2) = 94.63 ***	
Career/confidence	85	68.0 (60.0–76.4)	104	55.9 (48.4–63.1)	0.041
Ideals of beauty	7	5.6 (4.5–8.1)	29	15.6 (12.7–18.2)	0.011
Friends	20	16.0 (9.9–22.6)	31	16.7 (10.9–22.0)	0.996
Mention the impacts of CS		$x^{2}$(2) = 85.73 ***		$x^{2}$(2) = 196.20 ***	
Physical impacts	80	64.0 (55.5–71.9)	144	77.4 (72.1–83.9)	0.014
Psychological impacts	43	34.4 (25.9–42.5)	57	30.6 (24.2–37.9)	0.567
Social Impacts	10	8.0 (3.6–13.1)	15	8.1 (4.4–12.2	0.994

Note. *** *p* < 0.001, * *p* < 0.05.

## Data Availability

No new data were created or analyzed in this study. Data sharing is not applicable to this article.
